# Simulation Study of the Double-Gate Tunnel Field-Effect Transistor with Step Channel Thickness

**DOI:** 10.1186/s11671-020-03360-7

**Published:** 2020-06-15

**Authors:** Maolin Zhang, Yufeng Guo, Jun Zhang, Jiafei Yao, Jing Chen

**Affiliations:** College of Electronic and Optical Engineering and College of Microelectronics, Nanjing University of Posts and Telecommunications and National and Local Joint Engineering Laboratory of RF Integration and Micro-assembly Technology, Nanjing, China

**Keywords:** Tunnel field-effect transistors, Double-gate, Step channel thickness, Ambipolar current, TCAD simulation

## Abstract

Double-gate tunnel field-effect transistor (DG TFET) is expected to extend the limitations of leakage current and subthreshold slope. However, it also suffers from the ambipolar behavior with the symmetrical source/drain architecture. To overcome the ambipolar current, asymmetry must be introduced between the source and drain. In this paper, we investigate the performances of DG TFET with step channel thickness (SC TFET) by utilizing the 2D simulation. The asymmetry between source and drain is introduced through the step channel thickness; hence, the ambipolar behavior is expected to be relieved. The results show that the SC TFET exhibits significant reduction of ambipolar current compared with the conventional DG TFET. The mechanisms of SC TFET are thoroughly discussed to explore the physical insight. The impacts introduced by the structure parameters on onset voltage, subthreshold slope, drain current in on-state and ambipolar-state are also exhibited in determining the optimal structure.

## Background

As the extreme scaling process continues, CMOS technology with conventional MOSFET encounters various challenges such as the increasing leakage current and subthreshold slope (*SS*). Tunnel field-effect transistor (TFET), which utilizes the band-to-band tunneling (BTBT) mechanisms, is expected to extend the limitations of leakage current and *SS* [1–8]. Silicon-based TFET shows advantages such as high reliability and low cost. However, conventional silicon-based TFET exhibits a relatively low on-current in comparison with the MOSFET due to the constrained BTBT rate [9–11]. To develop the potential of silicon-based TFET, various novel TFET structures have been recently proposed to enhance the on-state current. The double-gate TFET (DG TFET) shows improved BTBT rate, leading to the enhanced on-current [12–14]. However, the ambipolar current of DG TFET is also increased since the BTBT rate improvement is activated in the ambipolar state as well [15]. To further overcome the ambipolar current, asymmetry must be introduced between source and drain [16]. DG TFETs with gate-drain underlap and less drain doping concentration are common methods to relieve the ambipolar problem [17–19]. But the gate-drain underlap requires greater S/D distance and less drain doping concentration increases the series resistances [15]. A previous work has shown that the ambipolar effects in the TFET with drain underlap could be further relieved by using the low-k spacers and by placing the contacts in the top and bottom configuration [15], suggesting that combined asymmetry strategies could be meaningful in improving the performance of the TFET. In our previous work, the FinFET with asymmetry fin width has been demonstrated to improve the performance of FinFET [20]. It is also believed that the channel thickness *t*_si_ has a significant impact on the BTBT rate of DG TFET [21]; hence, the asymmetry between the source thickness and the drain thickness might further relieve the ambipolar current and need to be studied thoroughly.

In this paper, we investigate the various performances of DG TFET with step channel thickness (SC TFET), the asymmetry between the source and drain is introduced through the step channel thickness so that the ambipolar current is expected to be reduced. The rest of this paper is arranged as follows: Section 2 presents the device structure and simulation setup. In Section 3, the mechanisms of the SC TFET is thoroughly discussed. The detailed discussion regarding the impacts of structure parameters on the transfer curves, onset voltage (*V*_onset_), average *SS* and drain current in on/ambipolar-state is also presented. Finally, the findings of this paper are enlightened in section IV.

## Structure and Simulation

**Fig. 1:**
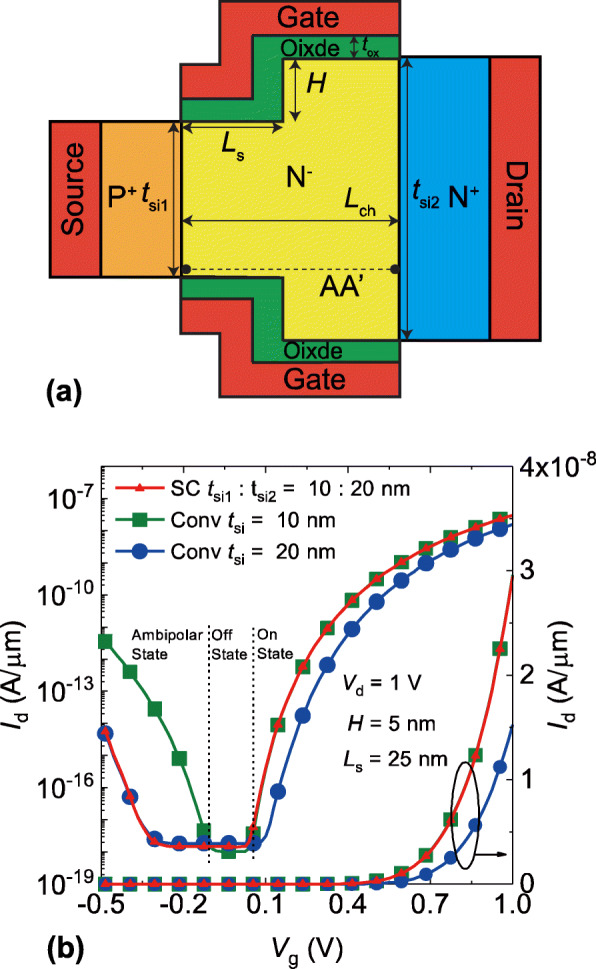
**a** 2D schematic diagram of the SC TFET, *t*si1, and *t*si2 are the channel thickness near the source region and the drain region, the asymmetry between the source and drain is obviously introduced. Cutline AA’ is the cutline along the horizontal direction. The vertical distance between the cutline and the surface of source region is 0.5 nm. **b** Transfer curves of the SC TFET and the conventional DG TFETs in log and linear scale

The schematic diagram of the SC TFET considered in this paper is shown in Fig. 1a. The channel thickness near the source region is not equal to the channel thickness near the drain region. The channel thickness changes stepwise at a certain point in the channel region. The step height and the step position are denoted as *H* and *L*_s_ respectively. *t*_si1_ and *t*_si2_ are the channel thickness near the source region and the drain region respectively. The effective oxide thickness (EOT) is 1 nm in our simulation. The source region is highly p-doped (10^20^ atoms/cm^3^) and the drain region is highly n-doped (10^20^ atoms/cm^3^) to reduce the series resistance [15], the channel region is lightly n-doped (10^17^ atoms/cm^3^). In order to analysis the onset voltage with various channel thickness setups, the work function of metal gate is fixed to 4.5 eV, the gate length is equal to the channel length *L*_ch_ and set to 50 nm [22–25]. The simulations are carried out using Sentaurus TCAD release I-2013.12 [26, 27]. The doping dependence model and the field dependent mobility model are Philips unified mobility model and Lombardi mobility model, respectively. The Fermi–Dirac statistics, Shockley–Read–Hall and Auger recombination model are also utilized. In order to account for the highly doped source/drain regions, the band gap narrowing model is activated. The nonlocal BTBT model based on Wentzel–Kramer–Brillouin (WKB) approximation tuned with the experimental results of [28] and the density-gradient quantization model are enabled to achieve the accurate simulation [29, 30]. The onset voltage is defined as the gate voltage at which the subthreshold slope is maximum. The average *SS* is extracted from the off-state current to *I*_d_ = 10^−11^ A/μm.

## Results and Discussion

### Transfer Curves and Mechanism

Figure 1b shows the transfer curves of SC TFET and conventional DG TFET in log and linear scale, respectively. We mark out the on-state, off-state, and ambipolar-state in Fig. 1b. For TFETs, a higher current of on-state and a lower current of ambipolar-state are always desired, which requires that the *V*_onset_ and *SS* should be low while the off-state should have a wide voltage range. As shown in Fig. 1b, the *V*_onset_ of conventional DG TFET with channel thickness of 10 nm are lower than that of the DG TFET with channel thickness of 20 nm. The extracted *V*_onset_ of DG TFET with *t*_si_ = 10 nm is 0.04 V and its extracted average *SS* is 44.8 mV/dec, the *V*_onset_ and the average *SS* of conventional DG TFET with *t*_si_ = 20 nm is 0.1 V and 50.6 mV/dec, respectively. The drain current of conventional DG TFET with *t*_si_ = 10 nm is improved by 94.7% compared to the conventional DG TFET with *t*_si_ = 20 nm. The main reason of this drain current improvement is the reduced *SS* and *V*_onset_. However, the off-state range of the conventional DG TFET with *t*_si_ = 10 nm is only 0.17 V. The conventional DG TFET with *t*_si_ = 20 nm, in comparison, exhibits off-state range of 0.45 V. As a result, the ambipolar-state current of the conventional DG TFET with *t*_si_ = 20 nm is reduced by 3 orders of magnitude compared to the conventional DG TFET with *t*_si_ = 10 nm.

For the fair comparison, the *t*_si1_ and *t*_si2_ of SC TFETs are equal to the channel thicknesses of above conventional DG TFETs, respectively. The narrower channel thickness *t*_si1_ of SC TFET is 10 nm and the wider channel thickness *t*_si2_ of SC TFET is 20 nm. The position of step is assumed at the middle of the channel and the *L*_s_ is 25 nm. One can observe that the SC TFET shows promising characteristics including the high drain current in the on-state as well as the wide range off-state. The drain current of SC TFET in the on-state is similar compared to the conventional DG TFET with *t*_si_ = 10 nm, the average *SS* is 45.8 mV/dec and the *V*_onset_ is 0.03 V. However, the off-state range of SC TFET is improved up to 123.5% and the ambipolar-state current is also reduced by 3 orders of magnitude in comparison with the conventional DG TFET with *t*_si_ = 10 nm. As a result, the on-state characteristics of SC TFET is similar to the conventional DG TFET with narrow channel thickness, the SC TFET also shows nearly parallel off/ambipolar curves to the conventional DG TFET with wide channel thickness. Hence, the SC TFET can achieve low *SS*, reduced *V*_onset_, and wide off-state range simultaneously.

**Fig. 2:**
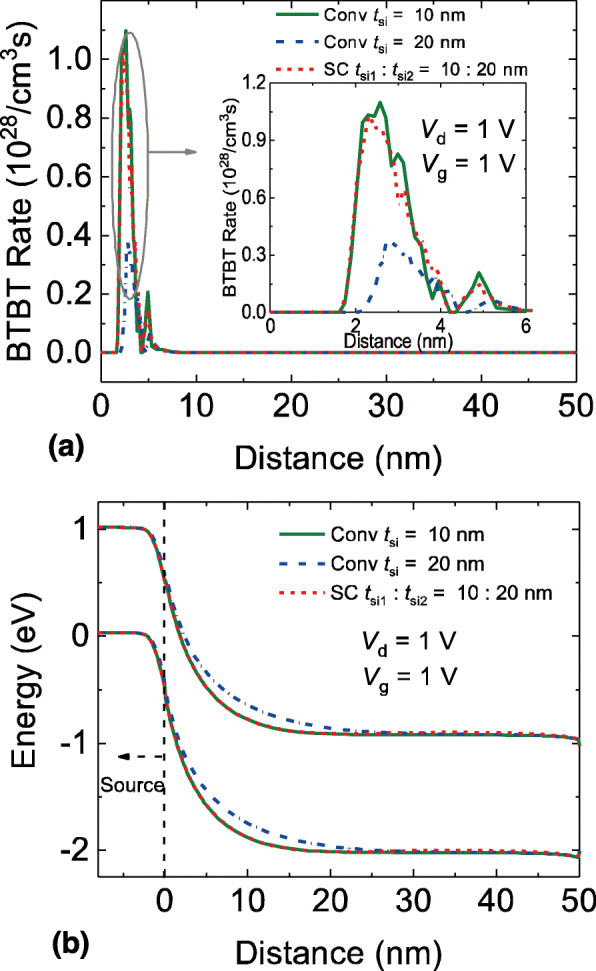
**a** BTBT rates and **b** energy band diagram of the SC TFET and the conventional DG TFETs in the on-state, distance is the lateral position of the cutline AA’ in Fig. 1

To explore the physical mechanism of the SC TFET, we compare the BTBT rates and energy band diagrams in the on-state, near onset point and ambipolar-state, respectively. Figure 2a shows the BTBT rates of the SC TFET and the conventional DG TFETs. It can be seen that the BTBT rate strongly depends on the channel thickness. In fact, the relationship between the channel thickness and the BTBT current *I*_BTBT_ can be expressed as [31].

1 $$ {I}_{BTBT}\propto \exp \left(-\frac{4\lambda \sqrt{2{m}^{\ast }{E_g}^{2/3}}}{3\mathrm{\hslash}\left(\Delta \Phi +{E}_g\right)}\right) $$

Where *λ* = (*ε*_si_*t*_si_*t*_ox_/2*ε*_ox_)^1/2^ is the natural length, *ε*_si_ and *ε*_ox_ are the silicon and oxide permittivity respectively and *t*_ox_ is the oxide thickness. Δ*Φ* is the energy range over which tunneling can take place, *E*_g_ is the band gap at the tunnel junction, and m* is the tunneling mass. Equation (1) indicates that the *I*_BTBT_ should increases as *t*_si_ reduces. Therefore, the BTBT rate of conventional DG TFET with *t*_si_ = 10 nm should greater than that of the conventional DG TFET with *t*_si_ = 20 nm. The SC TFET shows similar distribution of BTBT rate to the conventional DG TFET with *t*_si_ = 10 nm. This is because that the BTBT mainly occurs near the source junction in the on-state, hence the channel thickness near the source junction will determine the on-state BTBT rate. Figure 2b shows the energy band diagram of the SC TFET and the conventional DG TFETs. Since the BTBT rate is fundamentally related to the tunneling distance, the energy band diagram, which can present the tunneling distance clearly, will explain the origin of BTBT rates variation. In Fig. 2b, the minimum tunneling distance of the SC TFET is located near the source junction and is more or less equal to that of the conventional DG TFET with *t*_si_ = 10 nm. The minimum tunneling distance of conventional DG TFET with *t*_si_ = 20 nm is significantly wider; hence, its BTBT rate is reduced compared to the SC TFET and the conventional DG TFET with thinner channel thickness.

**Fig. 3:**
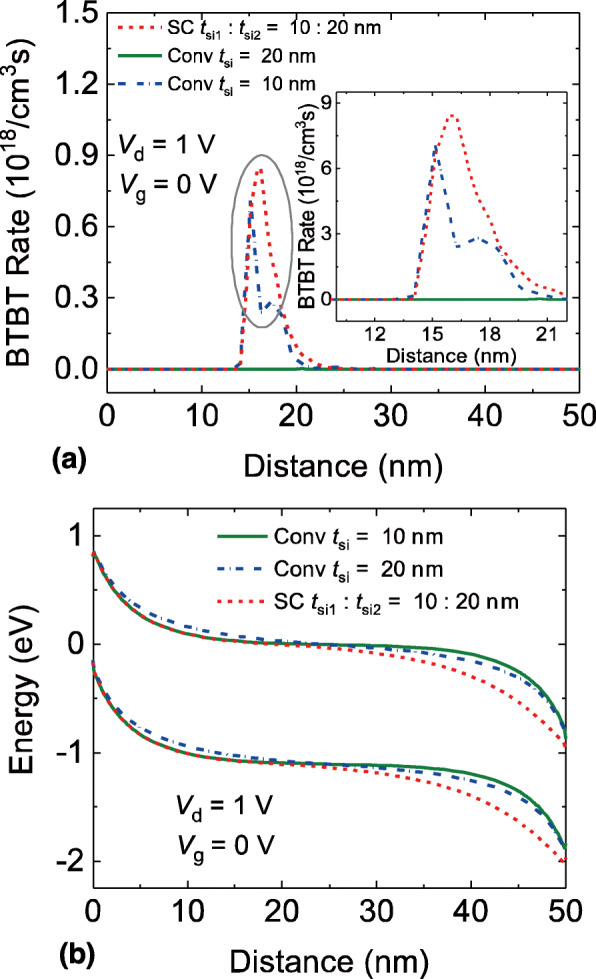
**a** BTBT rates and **b** energy band diagram of the SC TFET and the conventional DG TFETs in the near onset point, distance is the lateral position of the cutline AA’ in Fig. 1

Figure 3a shows the BTBT rates when the gate voltage is zero and is close to the onset voltage. It can be seen that the SC TFET owns the highest BTBT rate, followed by the conventional DG TFET with *t*_si_ = 10 nm. The conventional DG TFET with *t*_si_ = 20 nm shows the lowest BTBT rate. Figure 3b exhibits the corresponding energy band diagram. One can observe that the location of minimum distance from the valence band to the conduction band is at the center of channel region. Besides, the minimum distance of the conventional DG TFET with wider channel thickness is longer than that of the SC TFET and conventional DG TFET with thinner channel thickness. It indicates that the channel thickness also has a significant impact on the BTBT rate at the onset point. Hence, the *V*_onset_ is dependent on the channel thickness as well. Another fact is that the SC TFET exhibits greater BTBT rate than that of the conventional DG TFET with *t*_si_ = 10 nm even though their minimum channel thicknesses are equal. This phenomenon is attributed to the variation of potential distribution introduced by the corner of gate electrode, as can be also observed in other work [32, 33]. As a result, the SC TFET shows the lowest *V*_onset_, followed by the conventional DG TFET with thin channel thickness, and the conventional DG TFET with wide channel thickness exhibits the highest *V*_onset_.

**Fig. 4:**
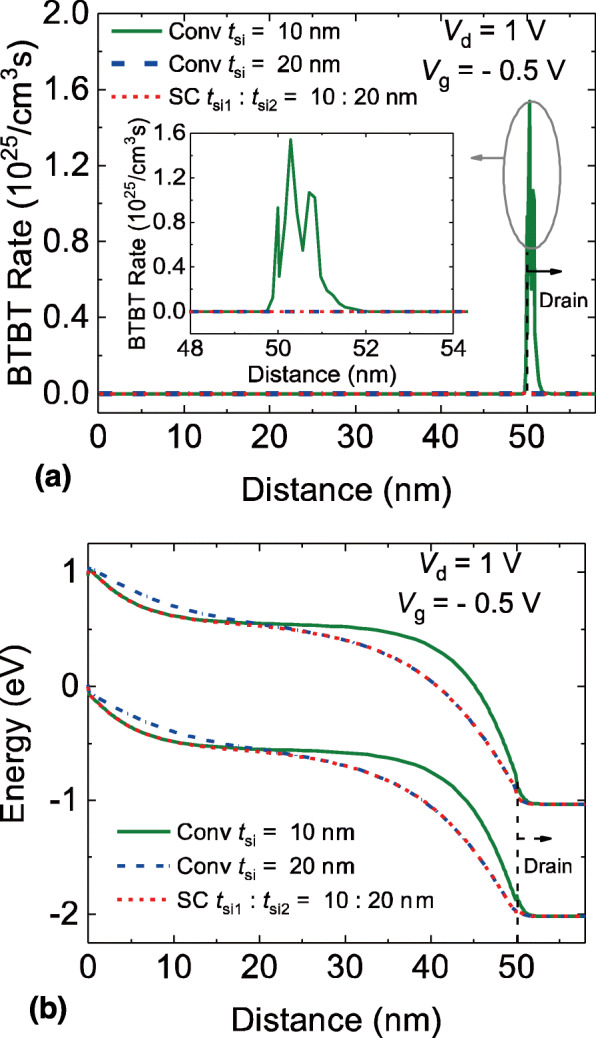
**a** BTBT rates and **b** energy band diagram of the SC TFET and the conventional DG TFETs in the ambipolar-state, distance is the lateral position of the cutline AA’ in Fig. 1

Figure 4a shows the comparison of BTBT rates in the ambipolar-state. Since the BTBT rate is strongly dependent on the channel thickness, the conventional TFET with *t*_si_ = 10 nm shows the most significant BTBT rate compared to the other two TFET structures. The SC TFET, however, shows the similar BTBT rate to the conventional DG TFET with *t*_si_ = 20 nm. It is because that the tunneling is mainly generated near the drain region and the SC TFET has wider channel thickness near the drain region. In Fig. 4b, the energy band diagram in the ambipolar-state is also exhibited. It can be clearly seen that the minimum tunneling distance is located near the drain region. Besides, the tunneling distances of SC TFET and conventional DG TFET with wider channel thickness are greater than that of the conventional DG TFET with thinner channel thickness, resulting in the low ambipolar current of SC TFET and conventional DG TFET with wider channel thickness.

### Impacts of H and L_S_ on DC Characteristics

**Fig. 5:**
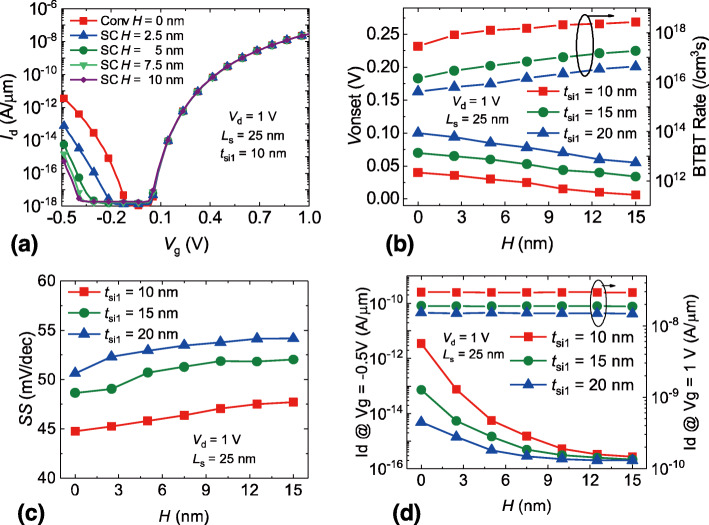
The impacts of *H* on the **a** transfer curves, **b***V*onset and BTBT rate, **c** average *SS*, and **d** drain current in on/ambipolar state, *H* is the height of the step and *H* = 0 nm represents the conventional DG TFET

Figure 5a shows the transfer curves of the SC TFET with various *H* and *t*_si1_ = 10 nm. It can be seen that the *H* has less impact on the on-state current. The ambipolar current, however, reduces significantly as the *H* increases, the off-state range improves with the rise of *H* as well. It can be also seen that the reduction of the ambipolar current decreases as the *H* increases. The reason for this is that the coupling effect of the double-gate structure tends to be less significant with a larger channel thickness [31]. Therefore, as the *H* increases, the BTBT rates become more independent of the channel thickness, leading to the saturation of ambipolar current. To further explore the optimal structure parameter, the effects of *H* varies from 0 to 15 nm on the device performances are extracted and shown in Fig. 5b–d. Figure 5b exhibits the *V*_onset_ and the BTBT rate variation with different *H* and *t*_si1_. It can be seen that the *V*_onset_ decreases monotonically with the increase of *H*. This is because that the corner of gate electrode would introduce the variation of the potential distribution in the channel region [32, 33], resulting in the alteration of the BTBT rate and the *V*_onset_. Figure 5b demonstrates that the BTBT rate increases as the *H* improves. As a result, the decreased *V*_onset_ can be found with the increased *H*. One can also observe that *V*_onset_ increases as the *t*_si1_ increases. The main reason is that the increased *t*_si1_ weakens the BTBT rate, resulting in a higher *V*_onset_. In Fig. 5c, the extracted average *SS* of SC TFET with various *H* and *t*_si1_ is shown. The trend of the *SS* with different *H* is opposite to that of the *V*_onset_. In another word, the *SS* rises as the *H* increases. We have mentioned that the on-state drain current is dependent on the *t*_si1_, so that the SC TFETs with different *H* but with the same *t*_si1_ should have the similar drain current in the on-state. Besides, it is also known that the *V*_onset_ decreases with the increase of *H*. This implies that the range of gate voltage to drive the same drain current is improved as the *H* increases. As a result, the average *SS* increases monotonically with the rise of *H*. It can be also seen that the increase of *t*_si1_ will undermine the *SS*, which is due to the reduced gate control capability. Figure 5d shows the drain current in the on-state and ambipolar-state with different *H* and *t*_si1_, respectively. The on-state current is nearly independent on the *H*, but it is greatly affected by the *t*_si1_, which corresponds to our previous result that the on-state tunneling mainly occurs near the source region and is strongly dependent on the channel thickness near the source region. The ambipolar current, however, reduces as the *H* increases. Since the ambipolar-state tunneling is dominated near the drain region, the increase of *H* will improve the channel thickness at the drain side and thus weakens the ambipolar current. It can be also seen that the ambipolar current drops more significantly when the *H* is less than 10 nm, which is due to the greater coupling effect with thinner channel thickness.

**Fig. 6:**
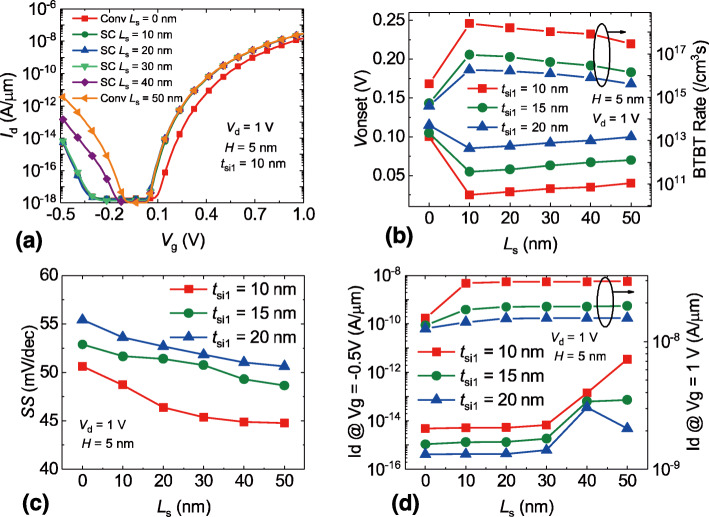
The impacts of *L*s on the **a** transfer curves, **b***V*onset and BTBT rate, **c** average *SS*, and **d** drain current in on/ambipolar state, *L*s is the lateral distance from the source region to the step, *L*s = 0 nm represents the conventional DG TFET with corresponding *t*si2 and *L*s = 50 nm represents the conventional DG TFET with corresponding *t*si1.

In Fig. 6a, the transfer curves of SC TFET with different *L*_s_ are presented respectively. *L*_s_ = 0 nm represents the conventional DG TFET with corresponding *t*_si2_ and *L*_s_ = 50 nm represents the conventional DG TFET with corresponding *t*_si1_. It can be seen that the location of step has a significant impact on the ambipolar current and the off-state range. The SC TFETs with *L*_s_ less than 30 nm show similar ambipolar current and off-state range. As the *L*_s_ exceeds 30 nm, the ambipolar current is greatly enhanced. Fig. 6b shows the *V*_onset_ and the BTBT rate variation with various *L*_s_ and *t*_si1_, the trend that the *V*_onset_ increases as the *t*_si1_ improves can be clearly observed as well. The change inflection point on the *L*_s_ = 10 nm is a result of the variety of the *t*_si1_. Since *L*_s_ = 0 nm is the conventional DG TFET with a larger channel thickness, the BTBT rate would reduce, leading to an increased *V*_onset_ and a decreased on-state current. Barring the case of conventional DG TFET, the *V*_onset_ of SC TFET is increased monotonically as the *L*_s_ rises, which is due to the reduced BTBT rate induced by the step channel structure. Figure 6c exhibits the impacts of the *L*_s_ and *t*_si1_ on the *SS* of SC TFETs. The increased *t*_si1_ results in the degraded *SS*. According to Eq. (1), a raised channel thickness would lower the coupling effects between the gate electrodes, leading to a reduced gate control capability and an increased *SS* [12]. As the *L*_s_ drops, the region with greater channel thickness will expand and would weaken the overall gate control capability. As a result, a reduced *L*_s_ will undermine the *SS* of SC TFETs, which can be clearly observed in Fig. 6c. Figure 6d presents the drain current in the on-state and ambipolar-state with different *L*_s_ and *t*_si1_, respectively. One can observe that the on-state current of SC TFET is more or less equal to the conventional DG TFET with corresponding *t*_si1_. As for the ambipolar current, the SC TFETs with *L*_s_ less than 30 nm show the similar current to the conventional DG TFETs with corresponding *t*_si2_. When the *L*_s_ increases to 40 nm, the ambipolar current rises dramatically. In fact, for the case of SC TFET with *L*_s_ = 40 nm and *t*_si1_ = 20 nm, its ambipolar current is even greater than that of the conventional DG TFET with *t*_si1_ = 20 nm. This is because that the vertical part of gate electrode can enhance the tunneling area especially when the vertical part of gate electrode is close to the PN junction [34]. It indicates that the *L*_s_ should be less than 40 nm for the purpose of reducing ambipolar current.

**Fig. 7:**
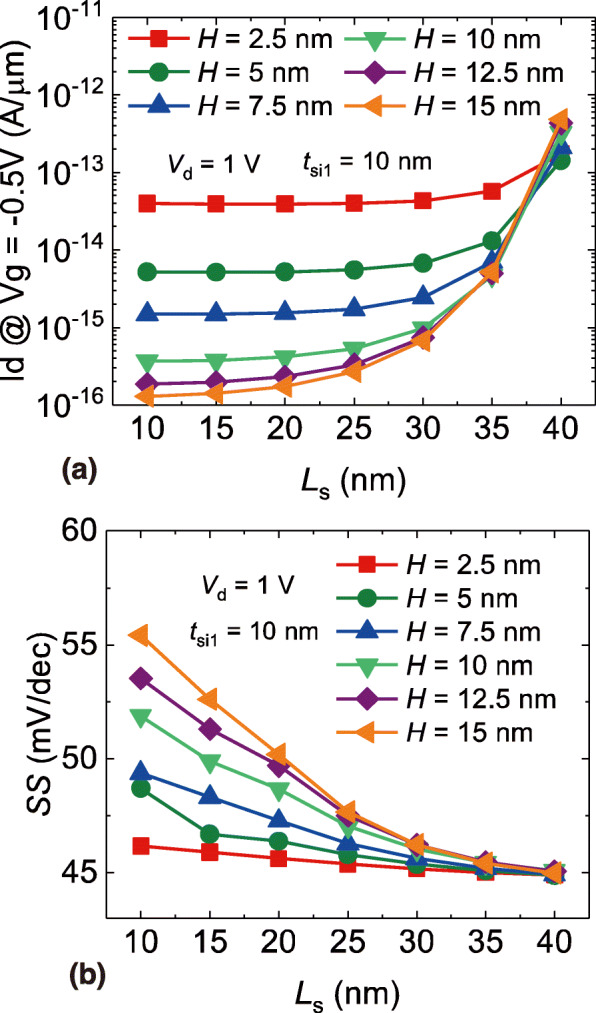
**a** The ambipolar current and **b** the subthreshold slope of the SC TFET as a function of the *L*S with various *H*

To determine the optimal structure parameters of the SC TFET, an orthogonal simulation is conducted by studying the combined effect of the *H* and the *L*_s_ on the device performance. The *t*_si1_ is fixed at 10 nm to achieve a greater on-state current. In Fig. 7a, the ambipolar current is extracted as a function of the *L*_s_ with various *H*. It can be clearly seen that the ambipolar current reduces significantly as the *H* decreases, which suggests that a higher *H* is promising in terms of achieving a lower ambipolar current. However, one can observe that the benefit from a greater *H* is less significant. Therefore, a *H* = 15 nm would be the optimal value considering that a larger *H* could only increase the device area. Meanwhile, a decreased *L*_s_ will also lower the ambipolar current especially with a greater *H*. Hence, a lower *L*_s_ is desired for the purpose of minimal ambipolar current. Nevertheless, a lower *L*_s_ could also lead to an increase of the subthreshold slope, as can be observed in Fig. 7b. The subthreshold slope increases slowly with a higher *L*_s_ but rises rapidly with a lower *L*_s_, indicating that a *L*_s_ about 25 nm would be the compromise value. As a result, the optimal device parameters would be *H* = 15 nm and *L*_s_ = 25 nm where both the ambipolar current and the subthreshold slope are relatively low.

### Fabrication Method

**Fig. 8:**
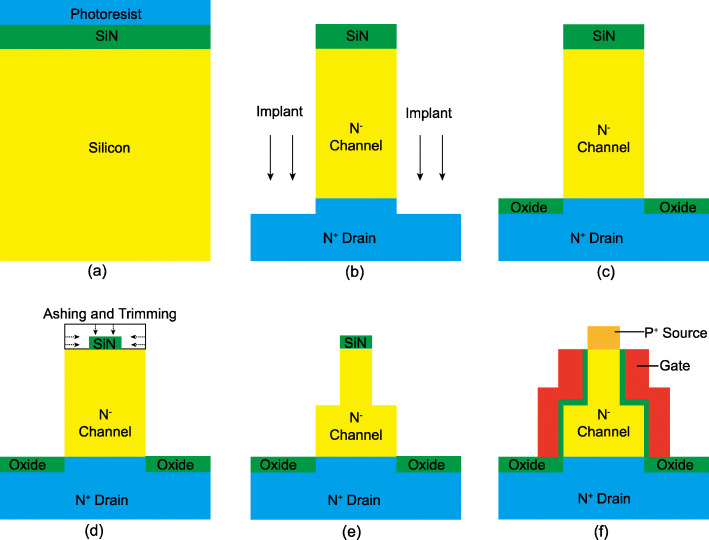
Fabrication process of the SC TFET. **a** Silicon substrate preparation with SiN and photoresist deposition. **b** Etching, implantation, and annealing. **c** Isolation oxide deposition. **d** Reducing the thickness and width of SiN by ashing and trimming. **e** The step channel thickness is introduced. **f** Gate oxide forming, gate deposition, gate planarization, and source region implantation

A feasible fabrication process of the SC TFET is exhibited in Fig. 8. Due to the unique shape of channel, the step channel thickness can be achieved more easily by adopting the vertical structure. The process begins by preparing the silicon substrate with SiN and photoresist deposition, as shown in Fig. 8a. In Fig. 8b, the SiN patterning is achieved by lithography, following by the etching to form the channel region, then the N^+^ region is introduced by a vertical As implantation and annealing [35]. After that, the isolation oxide is deposited to prevent the drain region from etching in the following process, as shown in Fig. 8c. In Fig. 8d, the ashing and trimming are adopted by utilizing the reaction ion etching [36] to reduce the thickness and width of SiN. The step channel thickness is then introduced by etching, as shown in Fig. 8e. The remain processes are similar to the conventional vertical TFET, involving gate oxide forming, gate deposition, silicon exposure, and source region implantation [35, 37], as shown in Fig. 8f.

## Conclusion

We investigate the electrical performances of DG TFET with step channel thickness (SC TFET) by utilizing the 2D simulation. The asymmetry between the source and drain is introduced through the step channel thickness; hence, the ambipolar behavior is significantly relieved. The SC TFET exhibits similar on-state characteristics of the conventional DG TFET with corresponding *t*_si1_ and parallel off/ambipolar curves of the conventional DG TFET with corresponding *t*_si2_. As a result, the SC TFET can achieve wide off-state range, low ambipolar current, and maintain the low *SS* simultaneously. The mechanisms of SC TFET are thoroughly discussed to explore the physical insight. The impacts introduced by the structure parameters on onset voltage, subthreshold slope, drain current in on-state, and ambipolar-state are also studied to determine the optimal structure. The SC TFET with *H* of 15 nm and *L*_s_ of 25 nm shows the optimal performances. Moreover, the architecture of step channel thickness provides an alternative asymmetry method. Since the combined asymmetry strategies are proved to be effective, our work could further provide performance improvement of the TFET.

## Data Availability

All data are fully available without restriction.

## Abbreviations

DG TFET: Double-gate tunnel field-effect transistor
SC TFET: DG TFET with step channel thickness
*SS*: Subthreshold slope
BTBT: Band-to-band tunneling
EOT: Effective oxide thickness
*V*_onset_: Onset voltage
*H*: Step height
*L*_s_: Step position
*L*_ch_: Channel length
*t*_si_: Channel thickness
*t*_si1_: Channel thickness near the source region
*t*_si2_: Channel thickness near the drain region
